# Diabetes Status and Association With Risk of Tuberculosis Among Korean Adults

**DOI:** 10.1001/jamanetworkopen.2021.26099

**Published:** 2021-09-21

**Authors:** Jung Eun Yoo, Dahye Kim, Kyungdo Han, Sang Youl Rhee, Dong Wook Shin, Hyun Lee

**Affiliations:** 1 Department of Family Medicine, Healthcare System Gangnam Center, Seoul National University Hospital, Seoul, Republic of Korea; 2 Department of Biostatistics, The Catholic University of Korea, Seoul, Republic of Korea; 3 Department of Statistics and Actuarial Science, Soongsil University, Seoul, Republic of Korea; 4 Department of Endocrinology and Metabolism, Kyung Hee University School of Medicine, Seoul, Republic of Korea; 5 Supportive Care Center, Department of Family Medicine, Samsung Medical Center, Sungkyunkwan University School of Medicine, Seoul, Republic of Korea; 6 Department of Clinical Research Design and Evaluation, Samsung Advanced Institute for Health Science and Technology, Sungkyunkwan University, Seoul, Republic of Korea; 7 Department of Digital Health, Samsung Advanced Institute for Health Science and Technology, Sungkyunkwan University, Seoul, Republic of Korea; 8 Division of Pulmonary Medicine and Allergy, Department of Internal Medicine, Hanyang University College of Medicine, Seoul, Republic of Korea

## Abstract

**Importance:**

The risk of tuberculosis (TB) associated with diabetes status, considering impaired fasting glucose or duration of diabetes, has not been well established.

**Objective:**

To evaluate the association of diabetes status with the development of TB in the general population.

**Design, Setting, and Participants:**

This population-based cohort study used data from the Korean National Health Insurance System database. Adult participants without a history of TB who underwent a health screening in 2009 were included. Eligible participants were followed up for incident TB cases from 1 year after the day of health screening until December 31, 2018. Data analysis was performed from September 2019 to September 2020.

**Exposures:**

Five levels of diabetes status were evaluated: normal glucose, impaired fasting glucose (considered as without diabetes), new-onset diabetes, diabetes duration less than 5 years, and diabetes duration 5 years or longer (considered as having diabetes).

**Main Outcomes and Measures:**

Newly diagnosed TB.

**Results:**

Among 4 423 177 participants, the mean (SD) age was 46.5 (13.9) years, and there were 2 597 142 men (58.7%). A total of 26 458 participants (0.6%) received a diagnosis of TB within a median (interquartile range) of 8.3 (8.1-8.6) years of follow-up. An increased risk of TB was observed in participants with diabetes compared with those without diabetes (adjusted hazard ratio [aHR], 1.48; 95% CI, 1.42-1.53). Although participants with impaired fasting glucose did not show an increased risk of TB incidence (aHR, 0.97; 95% CI, 0.93-1.01), the risk of TB incidence increased with diabetes duration (new-onset diabetes, aHR, 1.32; 95% CI, 1.23-1.42; diabetes duration <5 years, aHR, 1.45; 95% CI, 1.36-1.54; diabetes duration ≥5 years, aHR, 1.57; 95% CI, 1.48-1.66). Among participants with new-onset diabetes, compared with those in the lowest decile (fasting plasma glucose [FPG] level ≥126 but <128 mg/dL), the risk of TB was significantly increased for those in the highest decile (FPG level ≥202 mg/dL, aHR, 1.79; 95% CI, 1.42-2.26).

**Conclusions and Relevance:**

These findings suggest that longer diabetes duration is associated with development of TB, showing a dose-response association. Among participants with new-onset diabetes, incident TB was more common among those with FPG levels greater than or equal to 202 mg/dL.

## Key Points

**Question:**

Is the risk of developing tuberculosis (TB) associated with diabetes duration or fasting plasma glucose (FPG) levels?

**Findings:**

In this population-based cohort study, longer duration of diabetes was associated with the development of TB, showing a dose-response association. TB was more common among participants with FPG levels greater than or equal to 202 mg/dL.

**Meaning:**

These findings suggest that for TB prevention, FPG control should be reinforced even in patients with new-onset diabetes.

## Teaser

This cohort study evaluates the association of diabetes duration and fasting plasma glucose level with the development of tuberculosis in adults in Korea.

## Introduction

Tuberculosis (TB) continues to be a concern worldwide as a deadly communicable disease.^1^ The World Health Organization’s post-2015 global tuberculosis strategy calls for prioritizing interventions to address factors directly associated with TB risk, including diabetes.^2^ A synergic association between diabetes and TB has been suspected for decades but has emerged only recently as a global health concern, considering the increasing diabetes prevalence in TB-endemic regions.^3,4^

Diabetes appears to be associated with increased risk of lower respiratory tract infection, including TB,^3,4,5^ and to have a profound adverse effect on TB treatment outcomes.^6,7,8^ Even though TB is more associated with other immunosuppressive states, such as human immunodeficiency virus infection, because of the greater numbers, diabetes remains an important factor associated with TB incidence at the population level.^9^ A systematic review^4^ of 13 observational cohort studies revealed that diabetes was associated with a 3-fold increased risk of TB development. In addition, multidrug-resistant TB was reported to be associated with diabetes.^10^

Although several epidemiological studies^11,12,13,14,15,16,17^ have suggested that an association exists between diabetes status and the risk of TB development, they did not examine the effect of diabetes status considering impaired fasting glucose (IFG) and duration,^11,12,13,14,15,16^ and most of them did not fully adjust for potential confounding variables, such as lifestyle factors,^13,14,15^ socioeconomic status,^13,15^ or markers of chronic illness (eg, hemoglobin level).^12,14,15,16^ Additional limitations of previous studies include difficulties in applying study findings to the general public owing to specific populations studied, such as kidney transplant recipients^15^ or patients aged 65 years and older,^11^ as well as study design (eg, cross-sectional study^17^). Finally, despite the significant association between diabetes and development of TB, the association between fasting plasma glucose (FPG) level and future TB risk has not been elucidated in those with new-onset diabetes.

Therefore, in the present study, we aimed to investigate the risk of TB incidence according to diabetes status using a nationwide population-based database. We also aimed to evaluate the association between FPG level and TB risk among participants with new-onset diabetes.

## Methods

### Study Setting

This cohort study was designed and conducted according to the Strengthening the Reporting of Observational Studies in Epidemiology (STROBE) reporting guideline.^18^ This study was approved by Samsung Medical Center’s institutional review board. The institutional review board waived the need for consent from individual patients because the data used are public and anonymized under confidentiality guidelines.

In Korea, the National Health Insurance Service (NHIS) is the single insurer managed by the government and covers approximately 97% of the Korean population. The remaining 3% are covered by the Medical Aid program. The NHIS, which includes an eligibility database (eg, age, sex, socioeconomic variables, type of eligibility, and income level), medical treatment database (based on accounts submitted by medical service practitioners for medical expenses), health examination database (results of general health examinations and questionnaires on lifestyle and behavior), and a medical care institution database (types of medical care institution, location, equipment, and number of physicians), comprises a complete set of health information. Regarding the health examination database, the NHIS provides free biennial cardiovascular health screening to the entire Korean population aged 40 years and older and to all employees regardless of age. These services are also available annually for manual workers.

### Study Population

**Figure 1.**
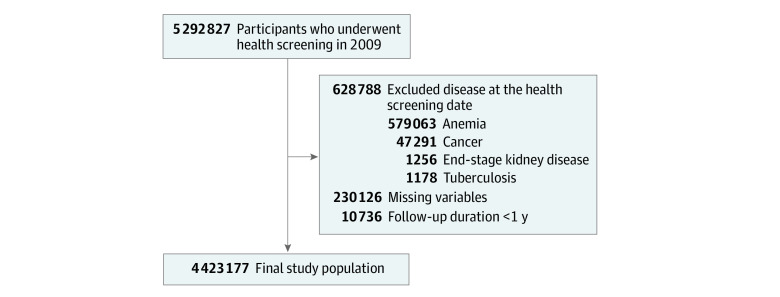
Flowchart of the Study Population

This study initially included a random sample (50%) of participants from all 5 292 827 adults older than 20 years who underwent a health screening in 2009. We excluded patients with a diagnosis of anemia (579 063 patients), cancer (47 291 patients), or end-stage kidney disease (1256 patients) at the health screening date; those with missing information (230 126 patients); those with a history of TB (1178 patients) or a new TB diagnosis (3109 patients); and those who died (7627 patients) within 1 year after the day of their health screening (Figure 1).

### Exposures: Diabetes Status

The presence of diabetes was defined as an *International Statistical Classification of Diseases and Related Health Problems, Tenth Revision (ICD-10) *codes E11 through E14 diagnosis, with at least 1 prescription of hypoglycemic agents before the health screening and FPG measurements at the health screening. This definition was based on the consensus of relevant findings widely used in previous studies.^19,20^ Regarding diabetes status at baseline, the participants were classified according to glycemic status into 5 categories: (1) normal glucose (FPG <100 mg/dL [to convert to millimoles per liter, multiply by 0.0555]), (2) IFG (FPG 100-125 mg/dL), (3) new-onset diabetes (FPG ≥126 mg/dL without diabetes diagnosis), (4) diabetes duration less than 5 years, and (5) diabetes duration 5 years or longer.

### Outcome: Incident TB

The study population was followed up from baseline (health screening date) to the date of TB diagnosis, censoring (eg, death), or the end of the study period (December 31, 2018), whichever came first. In this study, the primary end point was a new TB diagnosis, which was defined using the specific NHIS codes for TB (V206, V246, and V000). In Korea, the NHIS provides additional insurance coverage for all patients with TB (copayment reduction of up to 10%, compared with 20%-30% for other common diseases) to decrease the burden of TB in the nation, and those insurance codes specific for TB were automatically applied to patients with TB after confirmation of their diagnosis. Because the NHIS includes complete information on insured medical services throughout the country, the claims database enabled us to review all patients with active TB in the nation by means of their unique insurance codes.

### Covariates

Body mass index (BMI) was calculated as weight in kilograms divided by square of height in meters. Obesity was defined as BMI greater than or equal to 25, according to the Asia-Pacific BMI criteria established by the Western Pacific Region of the World Health Organization.^21^ Information on current smoking and alcohol consumption was obtained by questionnaire. Regular physical activity was defined as strenuous exercise for at least 20 minutes 3 times per week or moderate exercise for at least 30 minutes 5 times per week. Blood samples for measurement of serum hemoglobin, glucose, and cholesterol were obtained after an overnight fast. Hospitals where these health examinations were performed were certified by the NHIS and subjected to regular quality control.

Regarding comorbidities, the presence of dyslipidemia and chronic obstructive pulmonary disease (COPD) were determined according to *ICD-10* codes in the patients’ medical records. Information on the history of heart disease and stroke was collected by a self-administered questionnaire. The presence of chronic kidney disease was defined as a glomerular filtration rate less than 60 mL/min/1.73 m^2^, as estimated by the Modification of Diet in Renal Disease equation. The presence of COPD was based on *ICD-10* codes J43 through J44, except for J430, and the use of COPD drugs. Among participants with new-onset diabetes, FPG level was categorized by decile as follows: less than 128 mg/dL in decile 1 (the lowest decile), greater than or equal to 128 to less than 130 mg/dL in decile 2, greater than or equal to 130 to less than 133 mg/dL in decile 3, greater than or equal to 133 to 136 mg/dL in decile 4, greater than or equal to 136 to less than 140 mg/dL in decile 5, greater than or equal to 140 to less than 146 mg/dL in decile 6, greater than or equal to 146 to less than 154 mg/dL in decile 7, greater than or equal to 154 to less than 168 mg/dL in decile 8, greater than or equal to 168 to less than 202 mg/dL in decile 9, and greater than or equal to 202 mg/dL in decile 10 (the highest decile).

### Statistical Analysis

Analysis of variance and the χ^2^ test (all tests were 2-sided) were performed for continuous and categorical variables, respectively, to examine the differences among diabetes statuses. A Cox proportional hazards model was used to evaluate the association of diabetes status with TB incidence. We adjusted for age and sex in model 1. We adjusted for BMI, smoking, alcohol consumption, regular physical activity, income status (as quartiles), and plasma hemoglobin level in model 2. We also adjusted for comorbidities (glomerular filtration rate, ischemic heart disease, stroke, and COPD) in model 3. We also conducted stratified analyses of the association between diabetes status and TB incidence by sex and age (<45 years, 45-65 years, and ≥65 years), which might affect the association. Statistical analyses were performed using SAS statistical software version 9.4 (SAS Institute), and *P* < .05 was considered to indicate statistical significance. Data analysis was performed from September 2019 to September 2020.

## Results

### Baseline Characteristics

The final study population included 4 423 177 participants (mean [SD] age, 46.5 [13.9] years; 2 597 142 men [58.7%]). Table 1 shows their baseline characteristics according to glucose tolerance and diabetes status. The distribution of the total study cohort was as follows: normal glucose, 3 030 004 participants (68.5%); IFG, 1 017 410 participants (23.0%); new-onset diabetes, 135 448 participants (3.1%); diabetes duration less than 5 years, 128 882 participants (2.9%); and diabetes duration 5 years or longer, 111 433 participants (2.5%). The mean (SD) BMI was 23.8 (3.2). Participants with diabetes for 5 years or longer were more likely to be older, have obesity, have more comorbidities, perform regular physical activity, and be less likely to be current smokers or heavy consumers of alcohol than those in the other diabetes categories.

**Table 1.** Baseline Characteristics of the Study Population

Characteristic	Participants, No. (%)					
	Total (N = 4 423 177)	Normal glucose (n = 3 030 004)	Impaired fasting glucose (n = 1 017 410)	New-onset diabetes (n = 135 448)	Diabetes duration <5 y (n = 128 882)	Diabetes duration ≥5 y (n = 111 433)
Age, mean (SD), y	46.5 (13.9)	44.4 (13.7)	49.2 (13.1)	51.3 (12.4)	57.7 (10.9)	61.1 (9.9)
Sex						
Male	2 597 142 (58.7)	1 691 876 (55.8)	664 488 (65.3)	99 505 (73.5)	77 496 (60.1)	63 777 (57.2)
Female	1 826 035 (41.3)	1 338 128 (44.2)	352 922 (35.7)	35 943 (26.5)	51 386 (39.9)	47 656 (42.6)
Income						
Quartile 1 (lowest)	926 279 (20.9)	642 037 (21.2)	202 458 (19.9)	29 731 (22.0)	28 334 (22.0)	23 719 (21.3)
Quartile 2	776 029 (17.5)	551 584 (18.2)	167 973 (16.5)	22 590 (16.7)	18 793 (14.6)	15 089 (13.5)
Quartile 3	1 168 392 (26.4)	813 130 (26.8)	263 295 (25.9)	35 690 (26.4)	30 882 (24.0)	25 395 (22.8)
Quartile 4 (highest)	1552 477 (35.1)	1 023 253 (33.8)	383 684 (37.7)	47 437 (35.0)	50 873 (39.5)	47 230 (42.4)
Current smoker	1 255 154 (28.4)	847 905 (28.0)	302 371 (29.7)	49 338 (36.4)	32 164 (25.0)	23 376 (21.0)
Heavy consumer of alcohol	378 916 (8.6)	225 376 (7.4)	113 136 (11.1)	19 895 (14.7)	12 138 (9.42)	8371 (7.5)
Regular physical activity	806 316 (18.2)	529 130 (17.5)	195 012 (19.2)	25 761 (19.0)	28 570 (22.2)	27 843 (25.0)
Body mass index, mean (SD)^a^	23.8 (3.2)	23.4 (3.2)	24.5 (3.2)	25.1 (3.4)	25.5 (3.3)	24.8 (3.1)
Systolic blood pressure, mean (SD), mm Hg	122.7 (14.9)	120.8 (14.3)	126.0 (15.0)	130.1 (16.0)	128.9 (15.5)	129.0 (15.7)
Diastolic blood pressure, mean (SD), mmHg	76.6 (10.0)	75.6 (9.8)	78.6 (10.1)	80.9 (10.5)	79.4 (10.0)	78.1 (9.9)
Total cholesterol, mean (SD), mg/dL	196.0 (36.7)	193.3 (35.4)	202.8 (37.4)	209.0 (41.4)	196.7 (41.9)	190.3 (40.3)
Hemoglobin, mean (SD), g/dL	14.3 (1.3)	14.2 (1.3)	14.4 (1.3)	14.7 (1.3)	14.3 (1.3)	14.1 (1.2)
Comorbidities						
Dyslipidemia	808 596 (18.3)	426 208 (14.1)	228 978 (22.5)	39 157 (28.9)	62 598 (48.6)	51 655 (46.4)
Chronic kidney disease	367 925 (8.3)	219 209 (7.2)	98 323 (9.7)	14 246 (10.5)	17 126 (13.3)	19 021 (17.1)
Ischemic heart disease	83 768 (3.0)	42 911 (2.3)	24 443 (3.8)	3039 (3.4)	6345 (5.7)	7030 (6.8)
Stroke	44 797 (1.6)	23 279 (1.2)	13 916 (2.1)	1572 (1.7)	2830 (2.5)	3200 (3.1)
Chronic obstructive pulmonary disease	353 966 (8.0)	229 320 (7.6)	83 436 (8.2)	10 591 (7.8)	16 232 (12.6)	14 387 (12.9)
Antidiabetic drugs						
Metformin	180 017 (4.1)	NA	NA	NA	94 110 (73.0)	85 907 (77.1)
Sulfonylurea	184 449 (4.2)	NA	NA	NA	89 774 (69.7)	94 675 (85.0)
Meglitinide	10 344 (0.2)	NA	NA	NA	4401 (3.4)	5943 (5.3)
Thiazolidinedione	30 590 (0.7)	NA	NA	NA	13 954 (10.8)	16 636 (14.9)
Dipeptidyl peptidase-4 inhibitor	20 037 (0.5)	NA	NA	NA	10 819 (8.4)	9218 (8.3)
a-Glucosidase inhibitor	50 736 (1.2)	NA	NA	NA	17 763 (13.8)	32 973 (29.6)
Insulin	29 090 (0.7)	NA	NA	NA	10 267 (8.0)	18 823 (16.9)

Abbreviation: NA, not applicable.

SI conversion factors: To convert hemoglobin to grams per liter, multiply by 10; total cholesterol to millimoles per liter, multiply by 0.0250.

^a^ Body mass index is calculated as weight in kilograms divided by height in meters squared.

### Association of Diabetes Status With the Incidence of Tuberculosis

**Figure 2.**
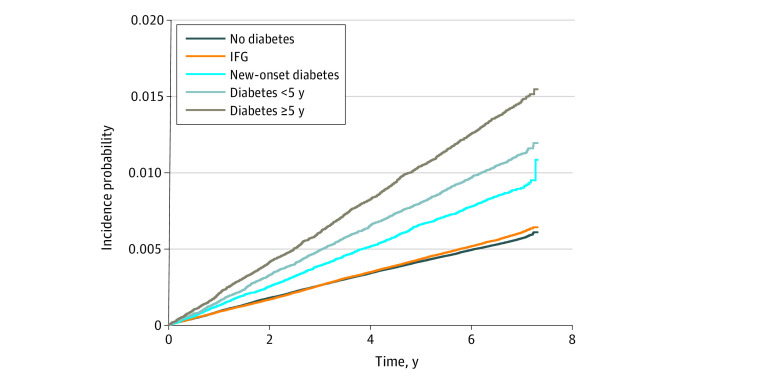
Cumulative Incidence of Tuberculosis According to Diabetes Status IFG indicates impaired fasting glucose.

The median (interquartile range) follow-up duration was 8.3 (8.1-8.6) years, and there were 26 458 new cases of TB (0.6% of the total participants). As shown in Figure 2, the cumulative incidence of TB was significantly different according to diabetes status (log-rank test, *P* < .001). The risk of TB was 48% higher in participants with diabetes compared with those without diabetes (adjusted hazard ratio [aHR], 1.48; 95% CI, 1.42-1.53) (Table 2). Although participants with IFG showed no increased risk of TB incidence (aHR, 0.97; 95% CI, 0.93-1.01), the risk of TB incidence was increased with diabetes duration (new-onset diabetes, aHR, 1.32; 95% CI, 1.23-1.42; diabetes duration <5 years, aHR, 1.45; 95% CI, 1.36-1.54; diabetes duration ≥5 years, aHR, 1.57; 95% CI, 1.48-1.66). After further consideration of the competing risks of death, the results remained consistent (eTable 1 in the Supplement).

**Table 2.** Incidence of Tuberculosis According to Diabetes Status

Variable	Participants, No.	Events, No.	Person-years, No.	IR per 1000 person-years	HR (95% CI)		
					Model 1^a^	Model 2^b^	Model 3^c^
Diabetes							
No	4 047 414	22 436	33 358 050.3	0.7	1 [Reference]	1 [Reference]	1 [Reference]
Yes	375 763	4022	3 008 706.4	1.3	1.98 (1.92-2.05)	1.52 (1.47-1.57)	1.48 (1.42-1.53)
Diabetes status							
Normal glucose	3 030 004	16 557	25 012 979.1	0.7	1 [Reference]	1 [Reference]	1 [Reference]
Impaired fasting glucose	1 017 410	5879	8 345 071.2	0.7	1.06 (1.03-1.10)	0.96 (0.93-0.99)	0.97 (0.93-1.01)
New-onset diabetes	135 448	1155	1 092 776.0	1.1	1.60 (1.50-1.69)	1.37 (1.29-1.46)	1.32 (1.23-1.42)
Diabetes duration, y							
<5	128 882	1360	1 037 085.4	1.3	1.98 (1.87-2.09)	1.51 (1.42-1.59)	1.45 (1.36-1.54)
≥5	111 433	1507	878 845.0	1.7	2.58 (2.45-2.72)	1.62 (1.53-1.71)	1.57 (1.48-1.66)

Abbreviations: HR, hazard ratio; IR, incidence rate.

^a^ Model 1 was adjusted for age and sex.

^b^ Model 2 was adjusted for age, sex, smoking status, alcohol consumption, regular physical activity, body mass index, and hemoglobin concentration.

^c^ Model 3 was adjusted for age, sex, smoking status, alcohol consumption, regular physical activity, body mass index, and hemoglobin concentration, estimated glomerular filtration rate, ischemic heart disease, stroke, and chronic obstructive pulmonary disease.

### Subgroup Analysis

Analyses stratified by age and sex were conducted. Diabetes duration 5 years or longer was associated with a higher incidence of TB in all subgroups compared with the group with normal glucose. These associations were prominent in men (aHR, 1.84; 95% CI, 1.72-1.98) and younger participants (aged <45 years, aHR, 4.61; 95% CI, 3.62-5.88) (eTable 2 and eTable 3 in the Supplement).

### Association Between FPG Level and TB Risk Among Participants With New-Onset Diabetes

**Figure 3.**
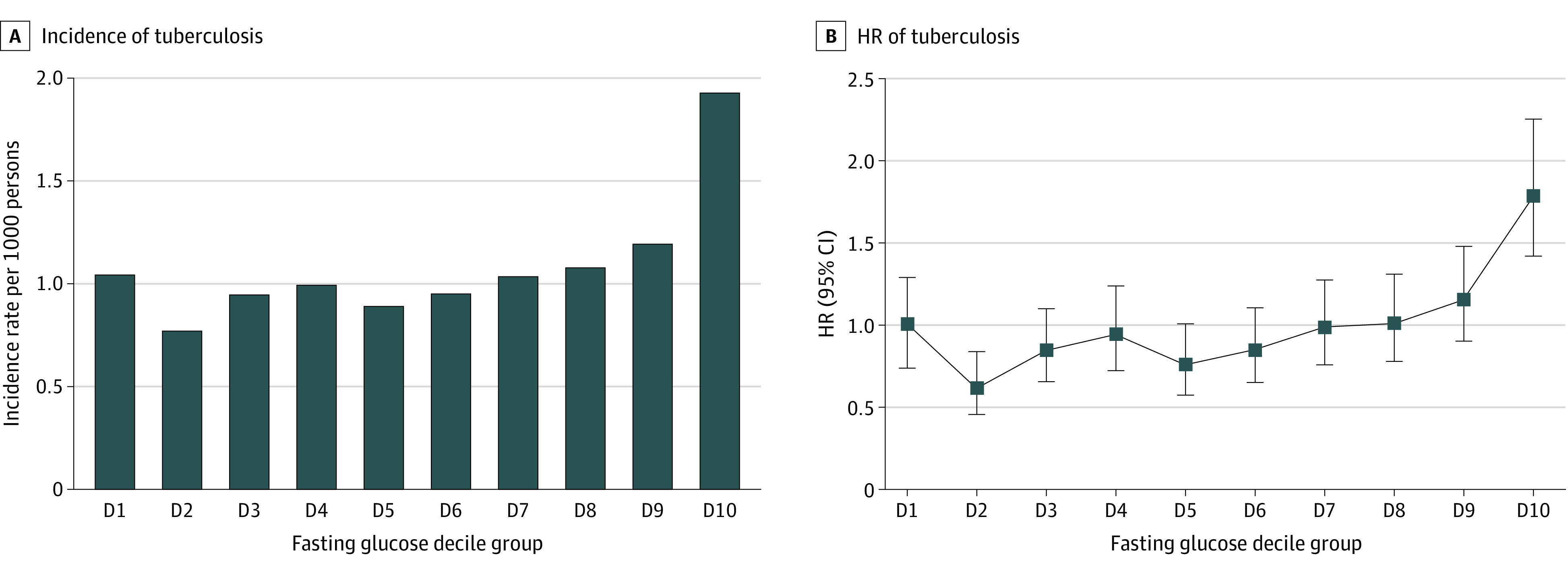
Incidence Rates and Hazard Ratios (HRs) of Tuberculosis by Decile (D) of Fasting Plasma Glucose (FPG) Level Among Patients With New-Onset Diabetes HRs were adjusted for age, sex, smoking status, alcohol consumption, regular physical activity, income, body mass index, and hemoglobin concentration. The range of FPG levels was less than 128 mg/dL (to convert to millimoles per liter, multiply by 0.0555) in D1, greater than or equal to 128 to less than 130 mg/dL in D2, greater than or equal to 130 to less than 133 mg/dL in D3, greater than or equal to 133 to less than 136 mg/dL in D4, greater than or equal to 136 to less than 140 mg/dL in D5, greater than or equal to 140 to less than 146 mg/dL in D6, greater than or equal to 146 to less than 154 mg/dL in D7, greater than or equal to 154 to less than 168 mg/dL in D8, greater than or equal to 168 to less than 202 mg/dL in D9, and greater than or equal to 202 mg/dL in D10.

The association between decile of FPG level and TB risk is depicted in Figure 3. Among participants with new-onset diabetes, compared with those in decile 1, the risk of TB was increased for those in decile 10 (aHR, 1.79; 95% CI, 1.42-2.26).

## Discussion

In this large-scale, population-based, cohort study investigating the association of diabetes status with risk of TB incidence, participants with diabetes had a 48% higher risk for TB incidence compared with those without diabetes. This association was robust in participants with longer diabetes duration, especially in men and younger participants. On the other hand, among participants without diabetes, those with IFG had no increased risk for TB. Among participants with new-onset diabetes, FPG level greater than or equal to 202 mg/dL was associated with an increased risk of TB.

The findings of this study support the positive association between diabetes duration and TB risk. The association between poor diabetic control, estimated by FPG and hemoglobin A_1c_, and increased risk of TB has been well demonstrated in previous studies.^11,22^ As a mechanism, hyperglycemia-induced impaired immunity, including phagocytic dysfunction and decreased T-helper 1–related cytokine response to *Mycobacterium tuberculosis*, has been suggested.^22,23,24^ Furthermore, T lymphocyte count is decreased in patients with diabetes.^25^ In addition to decreased T lymphocyte count, patients with diabetes and TB have reduced T cell function (eg, decreased T-helper 1 cytokine level, tumor necrosis factor–α production, and interleukin [IL]–1 and IL-6 production) compared with healthy control participants.^26^ Macrophages play an important role in defense against TB. In patients with diabetes, macrophage function, including phagocytic and bactericidal functions, is reduced.^27^ However, the effect of diabetes duration on TB risk has not been well established, and our study provides important implications that not only uncontrolled diabetes status but also diabetes duration can affect TB occurrence. There is a negative association between diabetes duration and insulin secretion.^28^ Also, reduced insulin receptor expression and downstream signaling in T lymphocytes have been shown in patients with diabetes.^29^ Because diminished insulin signaling includes reduced antigen-specific proliferation and proinflammatory cytokine production in T cells,^30^ decreased insulin production and altered insulin receptor–mediated signaling in T cells might contribute to increased TB risk in patients with diabetes of longer duration.

On the other hand, IFG was not associated with any change in the risk of incident TB, despite the large number sampled. In our study, BMI was lower in participants with normal glucose compared with those without IFG. However, we did not adjust for several comorbid conditions associated with low BMI (eg, chronic pulmonary diseases).^31^ It is well known that these comorbid conditions are associated with increased TB risk.^32^ Therefore, these comorbid conditions might be associated with increased TB risk through the mediation of low BMI, or they might act as confounding factors to increase TB prevalence in the population without diabetes compared with those with IFG. Those with IFG might have received more health interventions to prevent the development of diabetes, which might, in turn, reduce the risk of incident TB.

Our medical record review provided data showing the positive association between FPG level and TB risk among patients with new-onset diabetes. A previous study^22^ showed that patients with diabetes and FPG greater than 130 mg/dL had an approximately 2.2-fold increased risk of TB compared with those without diabetes. Although the previous study solely compared TB risk in patients with diabetes vs those without diabetes, our study also evaluated TB risk among patients with new-onset diabetes. Our study showed that among participants with new-onset diabetes, incident TB was more common among those with FPG levels greater than or equal to 202 mg/dL. However, as we only considered baseline FPG level and did not evaluate change in FPG after treatment, further studies are warranted. Nevertheless, it is likely that patients whose diabetes status progressed as a result of poor glucose control during the follow-up duration would have a higher risk of TB. Thus, it might be postulated that appropriate glucose control preventing the progression of IFG and early or poorly controlled diabetes could reduce the risk of TB.

The present study also showed that the association of diabetes duration 5 years or longer with TB risk was more robust in male and younger patients. Although the exact mechanism for this phenomenon is not fully explainable, testosterone could be a reason. Prior studies^33,34,35^ showed that testosterone plays a central role in sex-dependent susceptibility to TB. According to an animal study,^33^ orchidectomy after TB infection showed decreased bacilli burden and higher expression of tumor necrosis factor–α, IL-12, and interferon-γ. There is also evidence that testosterone is associated with diabetes status,^34^ and treatment with testosterone in hypogonadal men with type 2 diabetes showed improvement in insulin resistance and glycemic control.^35^ Accordingly, the interaction between testosterone, diabetes status, and TB risk might have led to the highest TB risk among male participants with longer diabetes duration. In a similar manner, there is a possibility that the association of age and diabetic duration with TB risk was mediated by age-related testosterone level. It is well known that testosterone level decreases with age in both male and female individuals.^36^

### Limitations

There are several potential limitations to be considered. First, we defined diabetes with health claims data, not clinical records. Therefore, diabetes quantification could be subject to the risk of under- or over-ascertainment. However, we used diagnosis code and medication records together, which have been shown to have high quantitative accuracy.^37^ Second, hemoglobin A_1c_ may be a more useful marker in patients with diabetes. However, this study was based on data that were not originally collected to study diabetes, and the national health screening program did not include hemoglobin A_1c_ measurements. Third, this study was restricted to include only those in the Korean population, where the incidence of TB is high and metabolic characteristics associated with diabetes could be different from those of patients of other ethnicities. Thus, future replicative research aimed at supporting our findings should include a variety of countries and ethnicities. Furthermore, because of the retrospective study design, the findings should be interpreted accordingly. For example, TB infection might worsen glycemic control^9^ or lead to IFG and new-onset diabetes.^38^ However, to minimize the possible effects of reverse causality, we excluded participants who received a TB diagnosis within 1 year of their health screening date.

## Conclusions

In this nationwide, population-based cohort study, we demonstrated a dose-response association between TB incidence and diabetic duration. These findings were more prominent in male participants and younger subgroups compared with the general study population. Regarding FPG level and TB risk, TB incidence was more common among patients with new-onset diabetes and FPG levels greater than or equal to 202 mg/dL.

## Notes

Supplement. **eTable 1.** Hazard Ratio (HR) and 95% Confidence Interval (CI) for Incidence of Tuberculosis According to Diabetes Status Considering Competing Risks of Death **eTable 2.** Hazard Ratio (HR) and 95% Confidence Interval (CI) for Incidence of Tuberculosis According to Diabetes Status by Sex **eTable 3.** Hazard Ratio (HR) and 95% Confidence Interval (CI) for Incidence of Tuberculosis According to Diabetes Status by Age
