# Supplementary material for: Diabetes Status and Association With Risk of Tuberculosis Among Korean Adults
Source: JAMA Netw Open. 2021 Sep 21;4(9):e2126099. doi: 10.1001/jamanetworkopen.2021.26099 (PMC8456384; doi:10.1001/jamanetworkopen.2021.26099)
Supplement: Supplement. — eTable 1. Hazard Ratio (HR) and 95% Confidence Interval (CI) for Incidence of Tuberculosis According to Diabetes Status Considering Competing Risks of Death eTable 2. Hazard Ratio (HR) and 95% Confidence Interval (CI) for Incidence of Tuberculosis According to Diabetes Status by Sex eTable 3. Hazard Ratio (HR) and 95% Confidence Interval (CI) for Incidence of Tuberculosis According to Diabetes Status by Age [file jamanetwopen-e2126099-s001.pdf]

# Supplemental Online Content

Yoo JE, Kim D, Han K, Rhee SY, Shin DW, Lee H. Diabetes status and association with risk of tuberculosis among Korean adults. *JAMA Netw Open*. 2021;4(9):e2126099. doi:10.1001/jamanetworkopen.2021.26099

**eTable 1.** Hazard Ratio (HR) and 95% Confidence Interval (CI) for Incidence of Tuberculosis According to Diabetes Status Considering Competing Risks of Death

**eTable 2.** Hazard Ratio (HR) and 95% Confidence Interval (CI) for Incidence of Tuberculosis According to Diabetes Status by Sex

**eTable 3.** Hazard Ratio (HR) and 95% Confidence Interval (CI) for Incidence of Tuberculosis According to Diabetes Status by Age

This supplemental material has been provided by the authors to give readers additional information about their work.

© 2021 Yoo JE et al. *JAMA Network Open.*

**eTable 1. Hazard Ratio (HR) and 95% Confidence Interval (CI) for Incidence of Tuberculosis According to Diabetes Status Considering Competing Risks of Death**

| Variable | HR (95% CI) | | |
|---|---|---|---|
| | Model 1[a] | Model 2[b] | Model 3[c] |
| **Diabetes** | | | |
| No | 1 [Reference] | 1 [Reference] | 1 [Reference] |
| Yes | **1.93 (1.87–2.00)** | **1.48 (1.43–1.53)** | **1.44 (1.39–1.50)** |
| **Diabetes status** | | | |
| Normal glucose | 1 [Reference] | 1 [Reference] | 1 [Reference] |
| Impaired fasting glucose | **1.06 (1.03–1.09)** | **0.96 (0.93–0.99)** | 0.97 (0.93–1.01) |
| New-onset diabetes | **1.57 (1.48–1.66)** | **1.34 (1.26–1.42)** | **1.30 (1.20–1.40)** |
| Diabetes duration <5 years | **1.92 (1.82–2.03)** | **1.47 (1.39–1.56)** | **1.42 (1.34–1.51)** |
| Diabetes duration ≥5 years | **2.48 (2.35–2.61)** | **1.57 (1.49–1.66)** | **1.53 (1.44–1.62)** |

HR, hazard ratio; CI, confidence interval.
[a]Model 1 was adjusted for age and sex.
[b]Model 2 was adjusted for age, sex, smoking status, alcohol consumption, regular physical activity, body mass index, and hemoglobin concentration.
[c]Model 3 was adjusted for age, sex, smoking status, alcohol consumption, regular physical activity, body mass index, and hemoglobin concentration, estimated glomerular filtration rate, ischemic heart disease, stroke, and chronic obstructive pulmonary disease.

© 2021 Yoo JE et al. *JAMA Network Open.*

**eTable 2. Hazard Ratio (HR) and 95% Confidence Interval (CI) for Incidence of Tuberculosis According to Diabetes Status by Sex**

| Sex | Variable | Total, No. | Event, No. | Person-years, No. | IR per 1000 person-years | HR (95% CI) | | |
|---|---|---|---|---|---|---|---|---|
| | | | | | | Model 1[a] | Model 2[b] | Model 3[c] |
| Men | **Diabetes** | | | | | | | |
| | No | 2,356,364 | 13,483 | 19,355,261.6 | 0.7 | 1 [Reference] | 1 [Reference] | 1 [Reference] |
| | Yes | 240,778 | 2,912 | 1,914,210.1 | 1.5 | **2.18 (2.10–2.27)** | **1.70 (1.63–1.78)** | **1.69 (1.61–1.77)** |
| | **Diabetes status** | | | | | | | |
| | Normal gluocse | 1,691,876 | 9,522 | 3,924,281.9 | 0.7 | 1 [Reference] | 1 [Reference] | 1 [Reference] |
| | Impaired fasting glucose | 664,488 | 3,961 | 5,430,979.7 | 0.7 | **1.07 (1.03–1.11)** | 1.00 (0.96–1.03) | 1.01 (0.96–1.05) |
| | New-onset diabetes | 99,505 | 948 | 799,921.8 | 1.2 | **1.73 (1.62–1.85)** | **1.55 (1.45–1.66)** | **1.53 (1.41–1.67)** |
| | Diabetes duration <5 years | 77,496 | 938 | 617,547.9 | 1.5 | **2.22 (2.07–2.37)** | **1.71 (1.60–1.83)** | **1.69 (1.56–1.82)** |
| | Diabetes duration ≥5 years | 63,777 | 1,026 | 496,740.3 | 2.1 | **3.02 (2.83–3.22)** | **1.86 (1.74–1.99)** | **1.84 (1.72–1.98)** |
| Women | **Diabetes** | | | | | | | |
| | No | 1,691,050 | 8,953 | 14,002,788.7 | 0.6 | 1 [Reference] | 1 [Reference] | 1 [Reference] |
| | Yes | 134,985 | 1,110 | 1,094,496.4 | 1.0 | **1.58 (1.49–1.68)** | **1.20 (1.12–1.28)** | **1.13 (1.05–1.21)** |
| | **Diabetes status** | | | | | | | |
| | Normal glucose | 1,338,128 | 7,035 | 1,088,697.3 | 0.6 | 1 [Reference] | 1 [Reference] | 1 [Reference] |
| | Impaired fasting glucose | 352,922 | 1,918 | 2,914,091.5 | 0.7 | 1.04 (0.99–1.09) | **0.91 (0.86–0.96)** | **0.92 (0.86–0.98)** |
| | New-onset diabetes | 35,943 | 207 | 292,854.2 | 0.7 | 1.11 (0.97–1.28) | 0.95 (0.83–1.09) | 0.87 (0.73–1.02) |
| | Diabetes duration <5 years | 51,386 | 422 | 419,537.5 | 1.0 | **1.58 (1.43–1.74)** | **1.20 (1.08–1.32)** | **1.11 (0.99–1.23)** |
| | Diabetes duration ≥5 years | 47,656 | 481 | 382,104.7 | 1.3 | **1.98 (1.80–2.17)** | **1.27 (1.15–1.39)** | **1.20 (1.09–1.33)** |

IR, incidence rate; HR, hazard ratio; CI, confidence interval.
[a]Model 1 was adjusted for age.
[b]Model 2 was adjusted for age, smoking status, alcohol consumption, regular physical activity, body mass index, and hemoglobin concentration.
[c]Model 3 was adjusted for age, smoking status, alcohol consumption, regular physical activity, body mass index, and hemoglobin concentration, estimated glomerular filtration rate, ischemic heart disease, stroke, and chronic obstructive pulmonary disease.

© 2021 Yoo JE et al. *JAMA Network Open.*

**eTable 3. Hazard Ratio (HR) and 95% Confidence Interval (CI) for Incidence of Tuberculosis According to Diabetes Status by Age**

| Age, y | Variable | Total, No. | Event, No. | Person-years, No. | IR per 1000 person-years | HR (95% CI) | | |
|---|---|---|---|---|---|---|---|---|
| | | | | | | Model 1[a] | Model 2[b] | Model 3[c] |
| <45 | **Diabetes** | | | | | | | |
| | No | 3,629,808 | 15,421 | 30,077,594.2 | 0.5 | 1 [Reference] | 1 [Reference] | 1 [Reference] |
| | Yes | 274,735 | 2,347 | 2,242,574.9 | 1.0 | **2.23 (2.03–2.45)** | **2.80 (2.54–3.08)** | **2.88 (2.57–3.23)** |
| | **Diabetes status** | | | | | | | |
| | Normal glcuose | 2,751,870 | 11,757 | 22,824,452.0 | 0.5 | 1 [Reference] | 1 [Reference] | 1 [Reference] |
| | Impaired fasting glucose | 877,938 | 3,664 | 7,253,142.2 | 0.5 | **0.86 (0.81–0.92)** | 0.97 (0.91–1.03) | 1.01 (0.93–1.09) |
| | New-onset diabetes | 114,504 | 834 | 934,845.5 | 0.9 | **1.94 (1.72–2.19)** | **2.40 (2.12–2.71)** | **2.40 (2.05–2.81)** |
| | Diabetes duration <5 years | 91,971 | 731 | 753,629.3 | 1.0 | **2.18 (1.82–2.61)** | **3.14 (2.61–3.77)** | **3.18 (2.59–3.91)** |
| | Diabetes duration ≥5 years | 68,260 | 782 | 554,100.1 | 1.4 | **3.72 (2.97–4.66)** | **4.60 (3.67–5.77)** | **4.61 (3.62–5.88)** |
| 45–65 | **Diabetes** | | | | | | | |
| | No | 3,629,808 | 15,421 | 30,077,594.2 | 0.5 | 1 [Reference] | 1 [Reference] | 1 [Reference] |
| | Yes | 274,735 | 2,347 | 2,242,574.9 | 1.0 | **2.04 (1.95–2.13)** | **1.90 (1.82–1.99)** | **1.68 (1.59–1.78)** |
| | **Diabetes status** | | | | | | | |
| | Normal gluocse | 2,751,870 | 11,757 | 22,824,452.0 | 0.5 | 1 [Reference] | 1 [Reference] | 1 [Reference] |
| | Impaired fasting glucose | 877,938 | 3,664 | 7,253,142.2 | 0.5 | 0.98 (0.95–1.02) | 0.97 (0.93–1.01) | 0.98 (0.92–1.04) |
| | New-onset diabetes | 114,504 | 834 | 934,845.5 | 0.9 | **1.73 (1.61–1.86)** | **1.68 (1.56–1.80)** | **1.41 (1.27–1.58)** |
| | Diabetes duration <5 years | 91,971 | 731 | 753,629.3 | 1.0 | **1.88 (1.75–2.03)** | **1.82 (1.69–1.97)** | **1.56 (1.42–1.72)** |
| | Diabetes duration ≥5 years | 68,260 | 782 | 554,100.1 | 1.4 | **2.74 (2.55–2.94)** | **2.25 (2.09–2.42)** | **1.98 (1.82–2.16)** |
| ≥65 | **Diabetes** | | | | | | | |
| | No | 417,606 | 7,015 | 3,280,456.2 | 2.1 | 1 [Reference] | 1 [Reference] | 1 [Reference] |
| | Yes | 101,028 | 1,675 | 766,131.5 | 2.2 | 1.03 (0.97–1.08) | **1.16 (1.09–1.22)** | **1.14 (1.07–1.21)** |
| | **Diabetes status** | | | | | | | |
| | Normal glucose | 278,134 | 4,800 | 2,188,527.2 | 2.2 | 1 [Reference] | 1 [Reference] | 1 [Reference] |
| | Impaired fasting glucose | 139,472 | 2,215 | 1,091,929.0 | 2.0 | **0.93 (0.88–0.97)** | 0.98 (0.93–1.03) | 0.96 (0.91–1.02) |
| | New-onset diabetes | 20,944 | 321 | 157,930.4 | 2.0 | 0.93 (0.83–1.04) | 0.98 (0.88–1.10) | 0.96 (0.84–1.10) |
| | Diabetes duration <5 years | 36,911 | 629 | 283,456.2 | 2.2 | 1.01 (0.93–1.10) | **1.22 (1.12–1.33)** | **1.18 (1.08–1.29)** |
| | Diabetes duration ≥5 years | 43,173 | 725 | 324,744.9 | 2.2 | 1.02 (0.95–1.11) | **1.18 (1.09–1.27)** | **1.15 (1.05–1.24)** |

© 2021 Yoo JE et al. *JAMA Network Open.*

IR, incidence rate; HR, hazard ratio; CI, confidence interval.
[a]Model 1 was adjusted for age and sex.
[b]Model 2 was adjusted for age, sex, smoking status, alcohol consumption, regular physical activity, body mass index, and hemoglobin concentration.
[c]Model 3 was adjusted for age, sex, smoking status, alcohol consumption, regular physical activity, body mass index, and hemoglobin concentration, estimated glomerular filtration rate, ischemic heart disease, stroke, and chronic obstructive pulmonary disease.

© 2021 Yoo JE et al. *JAMA Network Open.*
